# (*Z*)-2-(5-Acetyl-4-methyl-3-phenyl-2,3-dihydro-1,3-thia­zol-2-yl­idene)-3-(3-methyl-1-benzofuran-2-yl)-3-oxo­propane­nitrile

**DOI:** 10.1107/S1600536812035039

**Published:** 2012-08-15

**Authors:** Hoong-Kun Fun, Ching Kheng Quah, Hatem A. Abdel-Aziz, Hazem A. Ghabbour

**Affiliations:** a X-ray Crystallography Unit, School of Physics, Universiti Sains Malaysia, 11800 USM, Penang, Malaysia; b Department of Pharmaceutical Chemistry, College of Pharmacy, King Saud University, PO Box 2457, Riyadh 11451, Saudi Arabia

## Abstract

In the title compound, C_24_H_18_N_2_O_3_S, the benzofuran ring system (r.m.s. deviation = 0.010 Å) forms dihedral angles of 83.13 (17) and 8.92 (14)° with the benzene and thia­zole rings, respectively. The dihedral angle between the benzene and thia­zole rings is 84.51 (19)°. The mol­ecular structure features an intra­molecular C—H⋯O hydrogen bond, which closes an *S*(6) ring. There are no inter­molecular hydrogen bonds observed in this structure.

## Related literature

For background to and the biological activity of benzofuran derivatives, see: Abdel-Aziz *et al.* (2009) ▶; Abdel-Wahab *et al.* (2009 ▶). For further synthetic details, see: Dawood *et al.* (2005 ▶). For hydrogen-bond motifs, see: Bernstein *et al.* (1995 ▶). For related structures, see: Fun *et al.* (2012 ▶); Abdel-Aziz *et al.* (2012 ▶).
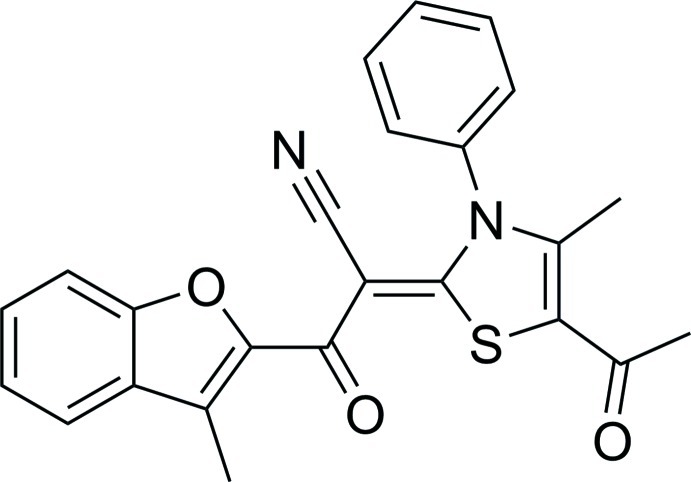


## Experimental

### 

#### Crystal data

C_24_H_18_N_2_O_3_S
*M* *_r_* = 414.46
Monoclinic, 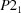

*a* = 9.7836 (4) Å
*b* = 6.3682 (3) Å
*c* = 16.2330 (6) Å
β = 100.351 (3)°
*V* = 994.92 (7) Å^3^
*Z* = 2
Cu *K*α radiation
μ = 1.69 mm^−1^
*T* = 296 K
0.92 × 0.09 × 0.06 mm

#### Data collection

Bruker SMART APEXII CCD diffractometer
Absorption correction: multi-scan (*SADABS*; Bruker, 2009 ▶) *T*_min_ = 0.306, *T*_max_ = 0.906
7151 measured reflections
2831 independent reflections
2310 reflections with *I* > 2σ(*I*)
*R*_int_ = 0.041

#### Refinement

*R*[*F* ^2^ > 2σ(*F* ^2^)] = 0.044
*wR*(*F* ^2^) = 0.119
*S* = 1.01
2831 reflections
275 parameters
1 restraint
H-atom parameters constrained
Δρ_max_ = 0.25 e Å^−3^
Δρ_min_ = −0.32 e Å^−3^
Absolute structure: Flack (1983 ▶), 831 Friedel pairs
Flack parameter: 0.03 (3)

### 

Data collection: *APEX2* (Bruker, 2009 ▶); cell refinement: *SAINT* (Bruker, 2009 ▶); data reduction: *SAINT*; program(s) used to solve structure: *SHELXTL* (Sheldrick, 2008 ▶); program(s) used to refine structure: *SHELXTL* ; molecular graphics: *SHELXTL*; software used to prepare material for publication: *SHELXTL* and *PLATON* (Spek, 2009 ▶).

## Supplementary Material

Crystal structure: contains datablock(s) global, I. DOI: 10.1107/S1600536812035039/hb6913sup1.cif

Structure factors: contains datablock(s) I. DOI: 10.1107/S1600536812035039/hb6913Isup2.hkl

Supplementary material file. DOI: 10.1107/S1600536812035039/hb6913Isup3.cml

Additional supplementary materials:  crystallographic information; 3D view; checkCIF report

## Figures and Tables

**Table 1:** Hydrogen-bond geometry (Å, °)

*D*—H⋯*A*	*D*—H	H⋯*A*	*D*⋯*A*	*D*—H⋯*A*
C14—H14*A*⋯O3	0.96	2.30	2.999 (5)	129

**Fig. 1.**
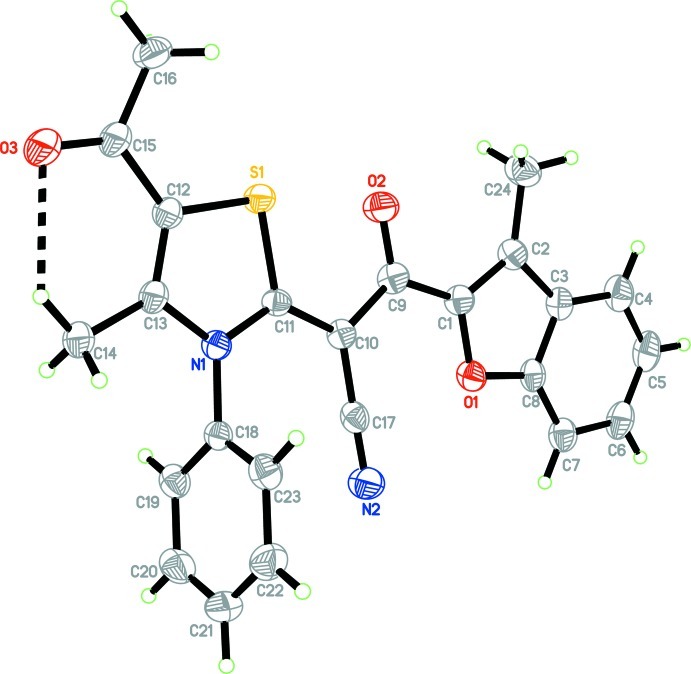
The molecular structure of the title compound showing 30% probability displacement ellipsoids for non-H atoms. Intramolecular hydrogen bond is shown as dashed line.

## supplementary crystallographic information

### Comment

In continuation of our interest in the structures and properties of benzofuran derivatives (Abdel-Aziz *et al.*, 2009; Abdel-Wahab *et al.*, 2009), we publish here the crystal structure of the title compound.

In the title molecule, Fig. 1, the benzofuran-2-yl ring system (O1/C1-C8, r.m.s. deviation = 0.010 Å) forms dihedral angles of 83.13 (17) and 8.92 (14)° with the benzene (C18-C23) and thiazol (S1/N1/C11-C13) rings, respectively. The dihedral angle between benzene and thiazol rings is 84.51 (19)°. Bond lengths and angles are comparable to related structures (Fun *et al.*, 2012; Abdel-Aziz *et al.*, 2012). The crystal structure is features an intramolecular C14–H14A···O3 hydrogen bond, forming an S(6) ring motif (Bernstein *et al.*, 1995).

There is no significant intermolecular hydrogen bond observed in this compound.

### Experimental

To a solution of 2-cyano-2-(3-methylbenzofuran-2-carbonyl)thioacetanilide (Dawood *et al.*, 2005), (0.33 g, 1 mmol) in ethanol (25 mL) and 3-chloropentane-2,4-dione (0.135 g, 1 mmol), triethylamine (0.2 mL) was added. The mixture was refluxed for 2 h, and then allowed to cool. The formed solid was filtered off, washed with ethanol, and recrystallized from EtOH/DMF solution to afford yellow needles of the title compound.

### Refinement

All H atoms were positioned geometrically and refined using a riding model with C–H = 0.93 or 0.96 Å and *U*_iso_(H) = 1.2 or 1.5 *U*_eq_(C). A rotating-group model was applied for the methyl groups. The reported Flack parameter was obtained by TWIN/BASF procedure in SHELXL (Sheldrick, 2008).

### Crystal data

**Table:**

C_24_H_18_N_2_O_3_S	*F*(000) = 432
*M**_r_* = 414.46	*D*_x_ = 1.383 Mg m^−^^3^
Monoclinic, *P*2_1_	Cu *K*α radiation, λ = 1.54178 Å
Hall symbol: P 2yb	Cell parameters from 1283 reflections
*a* = 9.7836 (4) Å	θ = 2.8–65.0°
*b* = 6.3682 (3) Å	µ = 1.69 mm^−^^1^
*c* = 16.2330 (6) Å	*T* = 296 K
β = 100.351 (3)°	Needle, yellow
*V* = 994.92 (7) Å^3^	0.92 × 0.09 × 0.06 mm
*Z* = 2	

### Data collection

**Table:**

Bruker SMART APEXII CCD diffractometer	2831 independent reflections
Radiation source: fine-focus sealed tube	2310 reflections with *I* > 2σ(*I*)
Graphite monochromator	*R*_int_ = 0.041
φ and ω scans	θ_max_ = 69.8°, θ_min_ = 2.8°
Absorption correction: multi-scan (*SADABS*; Bruker, 2009)	*h* = −11→11
*T*_min_ = 0.306, *T*_max_ = 0.906	*k* = −6→7
7151 measured reflections	*l* = −19→19

### Refinement

**Table:**

Refinement on *F*^2^	Secondary atom site location: difference Fourier map
Least-squares matrix: full	Hydrogen site location: inferred from neighbouring sites
*R*[*F*^2^ > 2σ(*F*^2^)] = 0.044	H-atom parameters constrained
*wR*(*F*^2^) = 0.119	*w* = 1/[σ^2^(*F*_o_^2^) + (0.0714*P*)^2^] where *P* = (*F*_o_^2^ + 2*F*_c_^2^)/3
*S* = 1.01	(Δ/σ)_max_ = 0.001
2831 reflections	Δρ_max_ = 0.25 e Å^−^^3^
275 parameters	Δρ_min_ = −0.32 e Å^−^^3^
1 restraint	Absolute structure: Flack (1983), 831 Friedel pairs
Primary atom site location: structure-invariant direct methods	Flack parameter: 0.03 (3)

### Special details

**Table:**

Geometry. All esds (except the esd in the dihedral angle between two l.s. planes) are estimated using the full covariance matrix. The cell esds are taken into account individually in the estimation of esds in distances, angles and torsion angles; correlations between esds in cell parameters are only used when they are defined by crystal symmetry. An approximate (isotropic) treatment of cell esds is used for estimating esds involving l.s. planes.
Refinement. Refinement of F^2^ against ALL reflections. The weighted R-factor wR and goodness of fit S are based on F^2^, conventional R-factors R are based on F, with F set to zero for negative F^2^. The threshold expression of F^2^ > 2sigma(F^2^) is used only for calculating R-factors(gt) etc. and is not relevant to the choice of reflections for refinement. R-factors based on F^2^ are statistically about twice as large as those based on F, and R- factors based on ALL data will be even larger.

### Fractional atomic coordinates and isotropic or equivalent isotropic displacement parameters (Å^2^)

**Table:**

	*x*	*y*	*z*	*U*_iso_*/*U*_eq_	
S1	0.23468 (9)	0.89038 (14)	0.13562 (4)	0.0454 (2)	
N1	0.1798 (2)	0.8805 (5)	0.28286 (14)	0.0386 (6)	
N2	0.3568 (3)	0.4048 (7)	0.39528 (18)	0.0667 (9)	
O1	0.4965 (2)	0.1891 (4)	0.26849 (14)	0.0482 (6)	
O2	0.3871 (3)	0.5930 (5)	0.11922 (15)	0.0618 (8)	
O3	−0.0119 (3)	1.3800 (6)	0.12668 (17)	0.0857 (11)	
C1	0.4888 (4)	0.3163 (6)	0.1977 (2)	0.0452 (8)	
C2	0.5710 (4)	0.2416 (6)	0.1451 (2)	0.0454 (8)	
C3	0.6340 (3)	0.0518 (6)	0.1842 (2)	0.0462 (9)	
C4	0.7255 (4)	−0.0986 (8)	0.1629 (2)	0.0600 (10)	
H4A	0.7602	−0.0871	0.1134	0.072*	
C5	0.7627 (4)	−0.2620 (7)	0.2159 (3)	0.0659 (12)	
H5A	0.8227	−0.3641	0.2020	0.079*	
C6	0.7126 (4)	−0.2797 (7)	0.2907 (3)	0.0615 (11)	
H6A	0.7400	−0.3935	0.3258	0.074*	
C7	0.6235 (3)	−0.1329 (7)	0.3142 (2)	0.0542 (9)	
H7A	0.5910	−0.1428	0.3645	0.065*	
C8	0.5854 (3)	0.0286 (6)	0.2590 (2)	0.0446 (8)	
C9	0.4003 (4)	0.5035 (6)	0.1875 (2)	0.0453 (8)	
C10	0.3304 (3)	0.5853 (6)	0.2518 (2)	0.0398 (7)	
C11	0.2520 (3)	0.7699 (5)	0.23210 (18)	0.0366 (7)	
C12	0.1271 (4)	1.0842 (6)	0.1664 (2)	0.0440 (8)	
C13	0.1085 (3)	1.0533 (6)	0.2467 (2)	0.0421 (8)	
C14	0.0248 (4)	1.1810 (7)	0.2967 (2)	0.0532 (10)	
H14A	−0.0085	1.3050	0.2659	0.080*	
H14B	0.0819	1.2204	0.3489	0.080*	
H14C	−0.0527	1.0996	0.3075	0.080*	
C15	0.0719 (4)	1.2547 (6)	0.1092 (2)	0.0514 (9)	
C16	0.1266 (4)	1.2735 (7)	0.0292 (2)	0.0591 (11)	
H16A	0.1020	1.4083	0.0044	0.089*	
H16B	0.0871	1.1649	−0.0087	0.089*	
H16C	0.2259	1.2593	0.0406	0.089*	
C17	0.3444 (3)	0.4875 (6)	0.3318 (2)	0.0446 (8)	
C18	0.1851 (3)	0.8306 (5)	0.37089 (19)	0.0377 (7)	
C19	0.0806 (4)	0.7114 (6)	0.3935 (2)	0.0488 (8)	
H19A	0.0069	0.6639	0.3535	0.059*	
C20	0.0879 (5)	0.6641 (7)	0.4774 (3)	0.0629 (11)	
H20A	0.0178	0.5853	0.4943	0.076*	
C21	0.1971 (5)	0.7323 (9)	0.5355 (2)	0.0687 (13)	
H21A	0.2022	0.6964	0.5915	0.082*	
C22	0.2995 (4)	0.8532 (8)	0.5121 (2)	0.0669 (13)	
H22A	0.3730	0.9003	0.5524	0.080*	
C23	0.2939 (3)	0.9058 (7)	0.4283 (2)	0.0496 (9)	
H23A	0.3621	0.9895	0.4118	0.059*	
C24	0.5966 (5)	0.3262 (8)	0.0633 (2)	0.0680 (12)	
H24D	0.6159	0.4739	0.0688	0.102*	
H24C	0.5157	0.3040	0.0211	0.102*	
H24B	0.6746	0.2552	0.0476	0.102*	

### Atomic displacement parameters (Å^2^)

**Table:**

	*U*^11^	*U*^22^	*U*^33^	*U*^12^	*U*^13^	*U*^23^
S1	0.0576 (5)	0.0388 (5)	0.0416 (4)	0.0113 (4)	0.0137 (3)	0.0032 (4)
N1	0.0428 (14)	0.0336 (15)	0.0403 (12)	0.0021 (14)	0.0101 (10)	0.0002 (14)
N2	0.088 (2)	0.057 (2)	0.0603 (18)	0.032 (2)	0.0280 (16)	0.015 (2)
O1	0.0559 (15)	0.0382 (15)	0.0538 (13)	0.0121 (11)	0.0188 (11)	−0.0001 (12)
O2	0.0834 (19)	0.0566 (19)	0.0504 (14)	0.0275 (16)	0.0252 (13)	0.0067 (14)
O3	0.103 (2)	0.080 (2)	0.0808 (19)	0.051 (2)	0.0368 (16)	0.034 (2)
C1	0.051 (2)	0.037 (2)	0.0476 (19)	0.0041 (15)	0.0099 (15)	−0.0022 (15)
C2	0.051 (2)	0.038 (2)	0.0480 (18)	0.0042 (16)	0.0122 (15)	−0.0086 (15)
C3	0.042 (2)	0.043 (2)	0.0524 (19)	0.0005 (15)	0.0056 (15)	−0.0141 (17)
C4	0.056 (2)	0.057 (3)	0.067 (2)	0.012 (2)	0.0123 (17)	−0.021 (2)
C5	0.056 (2)	0.050 (3)	0.089 (3)	0.021 (2)	0.005 (2)	−0.018 (2)
C6	0.052 (2)	0.041 (2)	0.087 (3)	0.0102 (18)	0.002 (2)	0.003 (2)
C7	0.048 (2)	0.046 (2)	0.069 (2)	0.0011 (19)	0.0105 (16)	0.001 (2)
C8	0.0388 (19)	0.035 (2)	0.060 (2)	0.0044 (15)	0.0093 (15)	−0.0044 (16)
C9	0.056 (2)	0.032 (2)	0.0478 (18)	0.0040 (17)	0.0091 (15)	−0.0048 (16)
C10	0.0428 (19)	0.0297 (18)	0.0475 (17)	−0.0001 (14)	0.0095 (14)	0.0002 (15)
C11	0.0422 (18)	0.0340 (19)	0.0343 (15)	−0.0012 (14)	0.0086 (13)	−0.0007 (13)
C12	0.049 (2)	0.037 (2)	0.0472 (18)	0.0065 (16)	0.0119 (15)	0.0031 (16)
C13	0.042 (2)	0.035 (2)	0.0489 (17)	0.0040 (14)	0.0060 (14)	0.0010 (15)
C14	0.060 (2)	0.046 (2)	0.055 (2)	0.0197 (19)	0.0148 (17)	0.0006 (19)
C15	0.057 (2)	0.040 (2)	0.057 (2)	0.0123 (17)	0.0098 (17)	0.0071 (17)
C16	0.072 (3)	0.052 (3)	0.052 (2)	0.008 (2)	0.0094 (18)	0.0144 (19)
C17	0.047 (2)	0.0349 (19)	0.055 (2)	0.0070 (16)	0.0174 (16)	−0.0003 (17)
C18	0.0425 (18)	0.0314 (19)	0.0407 (16)	0.0066 (13)	0.0112 (13)	0.0004 (13)
C19	0.049 (2)	0.042 (2)	0.058 (2)	0.0021 (17)	0.0161 (16)	0.0010 (18)
C20	0.072 (3)	0.050 (3)	0.075 (3)	0.008 (2)	0.036 (2)	0.020 (2)
C21	0.076 (3)	0.084 (4)	0.049 (2)	0.020 (3)	0.018 (2)	0.020 (2)
C22	0.066 (3)	0.082 (4)	0.0483 (19)	0.013 (3)	0.0006 (17)	−0.009 (2)
C23	0.0476 (19)	0.049 (2)	0.0528 (18)	−0.0024 (19)	0.0118 (15)	−0.008 (2)
C24	0.079 (3)	0.070 (3)	0.060 (2)	0.011 (2)	0.029 (2)	−0.002 (2)

### Geometric parameters (Å, º)

**Table:**

S1—C11	1.725 (3)	C10—C17	1.425 (4)
S1—C12	1.752 (4)	C12—C13	1.363 (4)
N1—C11	1.372 (4)	C12—C15	1.467 (5)
N1—C13	1.377 (5)	C13—C14	1.493 (4)
N1—C18	1.456 (4)	C14—H14A	0.9600
N2—C17	1.144 (4)	C14—H14B	0.9600
O1—C8	1.368 (4)	C14—H14C	0.9600
O1—C1	1.396 (4)	C15—C16	1.496 (5)
O2—C9	1.233 (4)	C16—H16A	0.9600
O3—C15	1.213 (5)	C16—H16B	0.9600
C1—C2	1.361 (5)	C16—H16C	0.9600
C1—C9	1.465 (5)	C18—C23	1.369 (5)
C2—C3	1.451 (5)	C18—C19	1.375 (4)
C2—C24	1.494 (5)	C19—C20	1.384 (5)
C3—C8	1.390 (5)	C19—H19A	0.9300
C3—C4	1.396 (5)	C20—C21	1.363 (6)
C4—C5	1.358 (6)	C20—H20A	0.9300
C4—H4A	0.9300	C21—C22	1.370 (6)
C5—C6	1.394 (5)	C21—H21A	0.9300
C5—H5A	0.9300	C22—C23	1.393 (5)
C6—C7	1.378 (5)	C22—H22A	0.9300
C6—H6A	0.9300	C23—H23A	0.9300
C7—C8	1.370 (5)	C24—H24D	0.9600
C7—H7A	0.9300	C24—H24C	0.9600
C9—C10	1.444 (4)	C24—H24B	0.9600
C10—C11	1.409 (5)		
			
C11—S1—C12	91.20 (16)	C12—C13—C14	128.5 (3)
C11—N1—C13	115.5 (3)	N1—C13—C14	119.3 (3)
C11—N1—C18	123.1 (3)	C13—C14—H14A	109.5
C13—N1—C18	121.3 (3)	C13—C14—H14B	109.5
C8—O1—C1	106.4 (2)	H14A—C14—H14B	109.5
C2—C1—O1	111.3 (3)	C13—C14—H14C	109.5
C2—C1—C9	128.1 (3)	H14A—C14—H14C	109.5
O1—C1—C9	120.6 (3)	H14B—C14—H14C	109.5
C1—C2—C3	105.6 (3)	O3—C15—C12	121.8 (3)
C1—C2—C24	130.1 (4)	O3—C15—C16	120.8 (4)
C3—C2—C24	124.3 (3)	C12—C15—C16	117.4 (3)
C8—C3—C4	118.7 (4)	C15—C16—H16A	109.5
C8—C3—C2	106.6 (3)	C15—C16—H16B	109.5
C4—C3—C2	134.7 (4)	H16A—C16—H16B	109.5
C5—C4—C3	118.6 (4)	C15—C16—H16C	109.5
C5—C4—H4A	120.7	H16A—C16—H16C	109.5
C3—C4—H4A	120.7	H16B—C16—H16C	109.5
C4—C5—C6	121.2 (4)	N2—C17—C10	178.4 (4)
C4—C5—H5A	119.4	C23—C18—C19	122.4 (3)
C6—C5—H5A	119.4	C23—C18—N1	118.6 (3)
C7—C6—C5	121.6 (4)	C19—C18—N1	119.0 (3)
C7—C6—H6A	119.2	C18—C19—C20	118.3 (4)
C5—C6—H6A	119.2	C18—C19—H19A	120.9
C8—C7—C6	116.2 (3)	C20—C19—H19A	120.9
C8—C7—H7A	121.9	C21—C20—C19	120.5 (4)
C6—C7—H7A	121.9	C21—C20—H20A	119.8
O1—C8—C7	126.3 (3)	C19—C20—H20A	119.8
O1—C8—C3	110.1 (3)	C20—C21—C22	120.5 (4)
C7—C8—C3	123.7 (3)	C20—C21—H21A	119.7
O2—C9—C10	119.8 (3)	C22—C21—H21A	119.7
O2—C9—C1	116.2 (3)	C21—C22—C23	120.3 (4)
C10—C9—C1	124.0 (3)	C21—C22—H22A	119.9
C11—C10—C17	122.1 (3)	C23—C22—H22A	119.9
C11—C10—C9	116.5 (3)	C18—C23—C22	118.1 (4)
C17—C10—C9	121.4 (3)	C18—C23—H23A	121.0
N1—C11—C10	127.6 (3)	C22—C23—H23A	121.0
N1—C11—S1	109.9 (2)	C2—C24—H24D	109.5
C10—C11—S1	122.6 (2)	C2—C24—H24C	109.5
C13—C12—C15	127.9 (3)	H24D—C24—H24C	109.5
C13—C12—S1	111.2 (3)	C2—C24—H24B	109.5
C15—C12—S1	120.9 (3)	H24D—C24—H24B	109.5
C12—C13—N1	112.3 (3)	H24C—C24—H24B	109.5
			
C8—O1—C1—C2	−0.7 (4)	C18—N1—C11—S1	−173.8 (2)
C8—O1—C1—C9	179.4 (3)	C17—C10—C11—N1	−0.2 (5)
O1—C1—C2—C3	0.9 (4)	C9—C10—C11—N1	−178.1 (3)
C9—C1—C2—C3	−179.2 (3)	C17—C10—C11—S1	179.8 (3)
O1—C1—C2—C24	−179.3 (4)	C9—C10—C11—S1	1.9 (4)
C9—C1—C2—C24	0.6 (7)	C12—S1—C11—N1	−1.1 (3)
C1—C2—C3—C8	−0.8 (4)	C12—S1—C11—C10	178.9 (3)
C24—C2—C3—C8	179.4 (4)	C11—S1—C12—C13	0.2 (3)
C1—C2—C3—C4	179.0 (4)	C11—S1—C12—C15	178.9 (3)
C24—C2—C3—C4	−0.8 (7)	C15—C12—C13—N1	−177.8 (3)
C8—C3—C4—C5	0.3 (6)	S1—C12—C13—N1	0.7 (4)
C2—C3—C4—C5	−179.4 (4)	C15—C12—C13—C14	1.6 (7)
C3—C4—C5—C6	−0.7 (6)	S1—C12—C13—C14	−179.9 (3)
C4—C5—C6—C7	0.0 (6)	C11—N1—C13—C12	−1.6 (4)
C5—C6—C7—C8	1.1 (6)	C18—N1—C13—C12	174.0 (3)
C1—O1—C8—C7	179.8 (3)	C11—N1—C13—C14	178.9 (3)
C1—O1—C8—C3	0.1 (4)	C18—N1—C13—C14	−5.4 (5)
C6—C7—C8—O1	178.8 (3)	C13—C12—C15—O3	−7.9 (7)
C6—C7—C8—C3	−1.6 (6)	S1—C12—C15—O3	173.7 (3)
C4—C3—C8—O1	−179.4 (3)	C13—C12—C15—C16	170.0 (4)
C2—C3—C8—O1	0.4 (4)	S1—C12—C15—C16	−8.4 (5)
C4—C3—C8—C7	0.9 (5)	C11—N1—C18—C23	82.6 (4)
C2—C3—C8—C7	−179.3 (3)	C13—N1—C18—C23	−92.7 (4)
C2—C1—C9—O2	8.2 (6)	C11—N1—C18—C19	−97.8 (4)
O1—C1—C9—O2	−172.0 (3)	C13—N1—C18—C19	86.9 (4)
C2—C1—C9—C10	−171.7 (4)	C23—C18—C19—C20	−1.0 (5)
O1—C1—C9—C10	8.2 (5)	N1—C18—C19—C20	179.5 (3)
O2—C9—C10—C11	−1.3 (5)	C18—C19—C20—C21	−0.8 (6)
C1—C9—C10—C11	178.5 (3)	C19—C20—C21—C22	1.7 (7)
O2—C9—C10—C17	−179.3 (3)	C20—C21—C22—C23	−0.8 (7)
C1—C9—C10—C17	0.6 (5)	C19—C18—C23—C22	1.9 (6)
C13—N1—C11—C10	−178.2 (3)	N1—C18—C23—C22	−178.6 (4)
C18—N1—C11—C10	6.2 (5)	C21—C22—C23—C18	−1.0 (6)
C13—N1—C11—S1	1.8 (4)		

### Hydrogen-bond geometry (Å, º)

**Table:**

*D*—H···*A*	*D*—H	H···*A*	*D*···*A*	*D*—H···*A*
C14—H14*A*···O3	0.96	2.30	2.999 (5)	129
